# Development and validation of the Japanese version of the Decisional Conflict Scale to investigate the value of pharmacists’ information: a before and after study

**DOI:** 10.1186/1472-6947-13-50

**Published:** 2013-04-17

**Authors:** Takashi Kawaguchi, Kanako Azuma, Takuhiro Yamaguchi, Hiroshi Soeda, Yusuke Sekine, Masayoshi Koinuma, Hironori Takeuchi, Takao Akashi, Sakae Unezaki

**Affiliations:** 1 Department of Practical Pharmacy, School of Pharmacy, Tokyo University of Pharmacy and Life Sciences, 1432-1 Horinouchi, Hachioji, Tokyo, Japan; 2 Department of Pharmacy, Tokyo Medical University Hospital, 6-7-1 Nishi-Shinjuku, Shinjuku, Tokyo, Japan; 3 Division of Biostatistics, Tohoku University Graduate School of Medicine, 1-1 Seiryo-machi, Aoba-ku, Sendai, Miyagi, Japan; 4 Laboratory of Clinical Pharmacy, Nihon University School of Pharmacy, 7-7-1 Narashinodai, Funabashi-shi, Chiba, Japan

**Keywords:** Cancer, Chemotherapy, Decisional conflict scale, Pharmacist

## Abstract

**Background:**

The information provided in patient-centered care and shared decision-making influences patients’ concerns and adherence to treatment. In the decision-making process, patients experience decisional conflict. The Decisional Conflict Scale (DCS) is a 16-item, self-administered questionnaire consisting of 5 subscales developed to assess patients’ decisional conflict. This study aimed to develop the Japanese version of the DCS and to clarify the influence of the information provided by pharmacists’ on decisional conflict among patients with cancer.

**Methods:**

We developed the Japanese version of the DCS by using the forward-backward translation method. One hundred patients who were recommended a new chemotherapy regimen were recruited. The psychometric properties of the Japanese DCS, including internal consistency, convergent validity, discriminant validity, and construct validity, were examined. We assessed the decisional conflict of patients before and after the pharmacists’ provision of information.

**Results:**

Ninety-four patients, predominately female, with an average age of 58.1 years were sampled. The scores on the 5 subscales of the DCS showed high internal consistency (Cronbach’s alpha = 0.84–0.96). Multi-trait scaling analysis and cluster analysis showed strong validity. The mean total DCS score decreased significantly from 40.2 to 31.7 after patients received information from the pharmacists (p < 0.001, paired t-test). Scores on all 5 subscales, namely, uncertainty, informed, values clarity, support, and effective decision, also significantly improved (p < 0.001 for all categories, paired t-test).

**Conclusions:**

The psychometric properties of the Japanese version of the DCS are considered appropriate for it to be administered to patients with cancer. Pharmacists’ provision of information was able to decrease decisional conflict among patients with cancer who were recommended a new chemotherapy regimen.

## Background

In recent years, patient-centered care and shared decision-making have been recognized as essential factors in oncology practice [1,2]. Because patient estimates of the risk/benefit of treatments vary, and because some therapeutic options have undesirable outcomes, patients with cancer may experience uncertainty and face difficult decisions [3-8]. Uncertainty and difficult options are likely to cause patients to experience decisional conflict [9]. O’Connor et al. defined a state of uncertainty concerning which course of action to take as decisional conflict and developed the Decisional Conflict Scale (DCS) to evaluate the decisional conflict that patients experience regarding the health care decision-making process [9].

The DCS is divided into five subscales of uncertainty, informed, values clarity, support, and effective decision-making, and is composed of 16 items that use a 5-point Likert-type response (i.e., strongly agree, agree, neither agree nor disagree, disagree, and strongly disagree). Decisional conflict was calculated from calculating the total scores obtained on these 16 items. The uncertainty subscale estimates the degree of uncertainty a patient faces in decision-making. Informed, values clarity, and support subscales are considered modifiable factors that contribute to uncertainty and represent feelings of being uninformed, the clarity of personal values, and feelings of being unsupported, respectively. The effective decision subscale measures the combination of informed choice, patient response value, and satisfaction. The total score and the score on each subscale are calculated according to the DCS user’s manual and both can range from 0 to 100, with 100 indicating extremely high decisional conflict. An effect size of 0.3 to 0.4 is assumed to be a meaningful difference [10]. A total score <25 is associated with decision implementation, while a total score ≥37.5 is associated with decision delay or indecision. Previous studies have indicated that decisions supporting intervention can ameliorate decisional conflict [10].

Understanding the information that a patient requires, particularly regarding the knowledge of treatment options, is essential in the decision-making process [11-13]. During diagnosis, nearly all patients require information on treatment, side effects, extent of disease, prognosis, and self-care [14]. Following treatment, patients would need information regarding treatment and recovery. Thus, these information needs and preferences can alter during the course of treatment [15]. In Japan, little research has been conducted on decisional conflict and patients’ perception of treatment in the decision-making process on patients with cancer. Medical paternalism, which is termed the “*omakase*” (entrusting) model in Japan, has existed in the Japanese physician-patient communication for many years [16]. This model, however, has been changing in the past decade. For example, Horikawa and colleagues reported that the percentage of information disclosure to patients with cancer had risen to 71% in the late 1990s from 27% in the early 1990s [17]. In response to these changes, there is a growing awareness among medical professionals that information provided to patients and patients’ attitudes toward participation in decision-making are essential to clinical practice in Japan [18,19]. In addition, patient satisfaction with information provided about medication affects their concern and adherence [20-22]. A responsibility of a pharmacist is to educate patients by providing treatment information [23]. A provision of information is a process of decision, and a decisional conflict is an outcome of decision. Therefore, we hypothesized that pharmacists’ provision of information regarding treatment would influence the decisional conflict in patients during decision-making. In addition, the development of instrument measuring decision outcome, the Japanese version of the DCS, is essential for assessment of pharmacists’ provision of information.

The aim of the present study was two-fold. First, we aimed to develop the Japanese version of the DCS and to examine the internal consistency, as well as the construct, criterion, and factorial validity in patients with cancer who were recommended a new chemotherapy regimen. Second, we aimed to analyze the influence of pharmacists’ provision of information on decisional conflict in patients with cancer.

## Methods

### Development of the Japanese version of the DCS

The DCS has three versions, namely, statement format, question format, and low literacy. The statement format (based on responses on statements with responses ranging from strongly agree to strongly disagree.), which is the one used in this study, has 16 items (e.g., “I am clear about which benefits matter most to me”) and five response categories. The statement format of the DCS was translated into Japanese through the forward-backward translation method [24]. Two native Japanese speakers conducted the forward translation. One of the translators was informed of the aim and concept of the questionnaire but the other was not. After the translations of both of the translators were synthesized, two translators back-translated the synthesized questionnaire into English. The two latter translators were native English language professionals without a medical background, and were not informed of the aim or concept of the questionnaire. After a pilot test to evaluate problems about semantic, idiomatic, and cognitive issues by using a hypothetical scenario of treatment choice with 40 health care providers, including physicians, nurses, pharmacists, and medical processors, we finalized the Japanese version of the DCS.

### Measurements

#### Decisional conflict

Each participant completed the Japanese version of the DCS before and after pharmacists’ provision of information. The repeated measurements result in random errors, which caused regression to the mean (RTM) [25]. Therefore, to reduce the effects of RTM, baseline decisional conflict was measured twice.

#### Baseline quality of life

This study included patients with various types of cancer and settings. Thus, participants may have various dysfunction and symptoms. Baseline quality of life (QOL) score, including assessment of function and symptoms, is known as a prognostic factor for survival in patients with cancer [26]. To assess the correlation between baseline QOL, a self-administered European Organisation for Research and Treatment of Cancer (EORTC) Quality of Life Questionnaire (QLQ)-C30 version 3.0 (30 items) was completed by the patients before the pharmacist provided information. The EORTC QLQ-C30 is composed of five functional scales (physical, role, emotional, cognitive, and social functioning), a global health status/QOL scale, three symptom scales (fatigue, pain, and nausea/vomiting), and six single items (dyspnea, insomnia, appetite loss, constipation, diarrhea, and financial difficulties). The validity and reliability of the Japanese version of EORTC QLQ-C30 has been verified in patients with cancer [27]. All scores were linearly transformed into scales of 0 to 100, according to the scoring manual. A higher functional scale score represented a more favorable status, while a higher symptom score indicated a poorer status.

#### Demographics and clinical information

We obtained demographic information, including age, gender, marital status, level of education, and employment status, from the patients through the questionnaire. The level of education was classified into three levels, namely, low (elementary: grades 1–6 and junior high school: grades 7–9), middle (high school: grades 10–12), and high (university or graduate school: grade 13 or above). Clinical information, such as cancer type, prior chemotherapy, and chemotherapy type, was obtained from the patients’ medical records.

#### Participants

This study was conducted on patients with cancer at Tokyo Medical University Hospital from June 2011 to April 2012. The criteria for eligibility were as follows: 20 to 75 years of age, diagnosed with any type of cancer, scheduled for receiving information from a pharmacist, and recommended for a new chemotherapy regimen. Patients who were not Japanese or had a serious psychiatric disease were excluded. Sample size was calculated to detect a meaningful difference of decisional conflict before and after the pharmacists’ provision of information. We estimated that 88 patients would be needed for an effect size of 0.3, a power of 80%, and a significance level of 0.05 (with a two-sided paired t-test). A target number of 100 registered patients was thus set to account for patients who may not complete the study.

#### Procedure

Data were collected through self-administered questionnaires before and after pharmacists’ provision of information. At Tokyo Medical University hospital, the therapeutic strategy was determined by physicians or a team conference that includes pharmacists. First, treating physicians met with patients and recommended the therapeutic strategy. Next, pharmacists met with patients and asked to complete the DCS and then left patients. At least one hour later, pharmacists returned and asked patients to complete the DCS a second time to address the RTM. After patients completed the survey for a second time, pharmacists provided information to patients for all treatment options. This information mainly consisted of the aim of treatment, side effects, and supportive care for the most highly recommended treatment option, and was relayed without a specific protocol. After the pharmacist has provided the necessary information, patients then completed the DCS a third time, the EORTC QLQ-C30, and the questionnaire regarding socio-demographics. Nine pharmacists (3 men and 6 women) were involved in this study. The mean (S.D.) age and work experience of these pharmacists were 30.8 (2.4) years and 7.6 (2.1) years, respectively. The mean length of discussion was 30 ± 11.8 min. After receiving information from the pharmacists’, the patients then decided about treatment with their physicians.

#### Analysis

Reliability and validity were investigated by using the average score on the first two DCSs to address RTM. The reliability of the five subscales was estimated by calculating Cronbach’s alpha. A Cronbach’s alpha value of >0.7 was considered acceptable. Construct validity, which refers to the ability of a measurement tool to actually measure the psychological concept being studied, was assessed through item-domain correlation and multi-trait scaling analysis for convergent validity and discriminant validity. Convergent validity tests whether constructs that should be related are related and discriminant validity tests whether believed unrelated constructs are, in fact, unrelated. Both are considered subcategories or subtypes of construct validity. A correlation of 0.4 or greater between an item and its domain, which must also be significantly higher than the correlation of the same item with a different domain, is required for convergent validity. Pearson correlation coefficient was calculated for the item-domain correlation to check the correlation of each item in a given scale for convergent validity and discriminant validity and for correlation between the score on each scale of the DCS and the scores on the EORTC QLQ-C30. Cluster analysis was conducted to examine the factorial structure of the DCS. We hypothesized that the optimal number of clusters was five, based on the DCS subscale number, and used SAS (version 9.1.3, SAS Institute Inc. Cary, North Carolina) VARCLUS procedure, which is a method for clustering variables. While the conventional clustering method divides observation into clusters, the VARCLUS produces readily interpretable disjoint clusters [27]. A paired t-test was used to analyze the change in DCS score before and after pharmacists’ provision of information. Descriptive statistics, including mean and standard deviation, were used to summarize patient demographics. Reliability and validity were analyzed using SAS, with the other statistics analyzed through JMP (version 9.02, SAS Institute Inc. Cary, North Carolina).

#### Ethical considerations

This study was conducted in accordance with the Japanese Ethical Guidelines for Epidemiological Research and World Medical Association Declaration of Helsinki. All patients gave written informed consent. The protocol was approved by the institutional review board of Tokyo Medical University.

## Results

### Participants

One hundred inpatients participated between June 2011 and April 2012. We excluded the data of six patients from analysis due to deviation from protocol; two were not administered the second DCS and four completed only one side of a double-sided questionnaire. Thus, 94 patients (94%) were ultimately included in the analysis. Participants’ basic characteristics are shown in Table 1. The average age of a patient was 58.1 ± 11.0 years with a range of 32–74 years. Female patients dominated the sample. Half of the patients were recommended for adjuvant chemotherapy and 27% of the patients had previously received chemotherapy treatment.

### Reliability

To assess internal consistency, Cronbach’s alpha coefficient was calculated. Internal consistency was high for all domains of the DCS (Cronbach’s alpha of 0.84–0.96) and satisfactory for all evaluated items (Table 2).

**Table 1:** Patient characteristics

		**Mean**	**SD**
*Age*			
	Total	58.1	11
	Men	62.9	8.4
	Women	55.1	11.5
		n	%
*Gender*			
	Men	37	39.4
	Women	57	60.6
*Type of cancer*			
	Gastroenterological	35	37.2
	Gynecological	31	33.0
	Lung	16	17.0
	Breast	8	8.5
	Other	4	4.3
*Type of chemotherapy*			
	Adjuvant	48	51.1
	Palliative	46	48.9
*Prior chemotherapy*			
	Yes	25	27.0
	No	69	73.0
*Marital status*			
	Married	67	71.3
	Unmarried	27	28.7
*Employment status*			
	Full-time	39	41.5
	Part-time	11	16.8
	Unemployed	44	51.7
*Education level*			
	High	45	47.8
	Middle	40	45.6
	Low	8	8.5
	Unknown	1	1.1

### Validity

Item-domain correlation was nearly equal for each item in the four subscales of uncertainty, informed, values clarity, and effective decision. The coefficient of item-domain correlation for item 8 and the support subscale (0.63) was relatively lower than that for items 7 (0.70) and 9 (0.80). The criterion of convergent validity and discriminant validity were met in all domains. The results of the cluster analysis are presented in Table 3. All items on the values clarity subscale were contained in Cluster 1 while Cluster 2 consisted of items on the uncertainty and effective decision subscales. Cluster 3 consisted of items 7, 9, and 13. Cluster 4 consisted of only item 8. Of items belonging to the informed domain, items 1 and 2 were grouped in Cluster 5 whereas item 3 (“I know the risk and side effects of each option”) was grouped in Cluster 1. The inter-cluster correlations of Clusters 1–2, 1–3, 1–5, 2–3, 2–5, and 3–5 were above 0.7. In contrast, the inter-cluster correlations that included Cluster 4 were relatively low (Cluster 1–4 [0.62], Cluster 2–4 [0.66], and Cluster 3–4 and 4–5 [0.64]).

**Table 2:** Internal consistency of Decisional Conflict Scale domains

Domain* (%)	**Item no.**	**Cronbach’s alpha**	**Item-domain correlation**	Convergent validity** (%)	Discriminant validity***
Uncertainty	10	0.91	0.83	100	100
	11		0.85		
	12		0.80		
Informed	1	0.90	0.82	100	100
	2		0.86		
	3		0.74		
Values clarity	4	0.96	0.90	100	100
	5		0.93		
	6		0.90		
Support	7	0.84	0.70	100	100
	8		0.63		
	9		0.80		
Effective decision	13	0.90	0.74	100	100
	14		0.70		
	15		0.82		
	16		0.82		

*Average score of the DCS before pharmacists’ provision of information.

**The percentage of items that passed the test of convergent validity.

***The percentage of items that passed the test of discriminant validity.

### Correlation between DCS and QOL

With respect to baseline QOL, the mean (SD) of global health status (GHS)/QOL was 55.1 (23.2). All Spearman correlation coefficients between each DCS score and the EORTC QLQ-C30, including GHS/QOL, function scores, and symptom scores, were not above 0.4; therefore, no correlations were observed between decisional conflict and QOL.

### Influence of pharmacists’ provision of information

The mean (SD) of the total score at baseline, 40.2 (21.1), was significantly reduced to 31.7 (20.5) after factoring in pharmacists’ provision of information (p < 0.001, paired t-test) (Table 4). Similarly, the scores on the subscales of uncertainty (45.0 vs. 37.2; difference: -8.5, p < 0.0001), informed (40.3 vs. 31.9; difference: -7.8, p < 0.0001), values clarity (44.2 vs. 32.1; difference: -12.1, p < 0.0001), support (37.2 vs. 29.4; difference: -7.8, p < 0.0001), and effective decision (35.7 vs. 28.7; difference: -7.0, p < 0.0001) decreased significantly. A similar trend was observed across pharmacists. The standardized response mean and effect size of the total score were 0.6 and 0.4, respectively.

## Discussion

In this study, we evaluated the psychometric properties of the Japanese version of the DCS and the influence of pharmacists’ information on decisional conflict among patients with cancer who were recommended to initiate a new chemotherapy regimen. The Japanese version of the DCS showed high reliability and validity and was influenced by pharmacists’ provision of information.

**Table 3:** Cluster structure of Decisional Conflict Scale items

**Domain**	**Item no.**	**Cluster structure**				
		**Cluster 1**	**Cluster 2**	**Cluster 3**	**Cluster 4**	**Cluster 5**
Uncertainty	10	0.79	**0.89**	0.79	0.68	0.77
	11	0.66	**0.90**	0.72	0.60	0.63
	12	0.61	**0.89**	0.61	0.46	0.57
Informed	1	0.78	0.65	0.72	0.63	**0.96**
	2	0.83	0.70	0.75	0.60	**0.96**
	3	**0.87**	0.54	0.63	0.50	0.74
Values clarity	4	**0.94**	0.76	0.80	0.60	0.83
	5	**0.96**	0.73	0.76	0.60	0.79
	6	**0.94**	0.69	0.78	0.60	0.75
Support	7	0.69	0.60	**0.88**	0.53	0.65
	8	0.62	0.66	0.64	**1.00**	0.64
	9	0.71	0.71	**0.93**	0.66	0.73
Effective decision	13	0.76	0.79	**0.89**	0.54	0.69
	14	0.56	**0.79**	0.54	0.57	0.51
	15	0.65	**0.89**	0.75	0.65	0.59
	16	0.60	**0.90**	0.67	0.55	0.63
Variation explained^*^ (%)		86.3	77.3	81.3	100.0	91.9
	Inter-Cluster		Cluster 1-2	Cluster 1-3	Cluster 1-4	Cluster 1-5
	Correlation		0.73	0.80	0.62	0.84
				Cluster 2-3	Cluster 2-4	Cluster 2-5
				0.78	0.66	0.70
					Cluster 3-4	Cluster 3-5
					0.64	0.77
						Cluster 4-5
						0.64

Correlation coefficients between each item and each cluster component.

Bold characters indicate the highest correlation coefficients.

*Total variation explained = 83.5%.

The Japanese version of the DCS has acceptable convergent and discriminant validity and acceptable internal consistency. However, regarding construct validity, items of the Japanese version of the DCS were not clustered similar to the original version of the DCS. In particular, items of uncertainty and effective decision were in the same cluster. There are several potential causes for this dissimilarity. Firstly, half of the participants were recommended to receive adjuvant chemotherapy. Generally, physicians strongly recommend patients receive adjuvant chemotherapy; however, patients in palliative settings have the added option of watchful waiting [28]. Hence, almost all inpatients recommended to receive adjuvant chemotherapy in this study may have been close to not having a choice of no treatment. Koedoot et al. have described the problem with using the effective decision subscale in the decision-making process with these cases [29]. Because we aimed to evaluate the effect of the pharmacists’ provision of information on decision-making, decisional conflict was assessed only after physician counseling. Thus, the results of the cluster analysis may be affected by the timing of the administration of the DCS. Secondly, our study included patients with different types of cancer (i.e., breast, lung, or gynecological) and settings (i.e., adjuvant or palliative). The number of treatment options and need for chemotherapy usually depend on the type of cancer and settings, which would affect decisional conflict. Thus, the results of cluster analysis may reflect this treatment-related decisional conflict. Thirdly, we did not assess patient preference for decision control. The patients’ role in a decision is associated with their decision characteristics and differs according to evidence of treatment, treatment modality, and palliative settings [30]. Indeed, Watanabe and colleagues found that the preferences of Japanese patients with cancer varied with their role in decision making, similar to patients in Western countries [19]. Therefore, future studies could consider the relationship between preferred role and effective decision through the Control Preference Scale developed by Degner et al [31].

**Table 4:** Differences in Decisional Conflict Scale domain scores between pre- and post-pharmacists’ provision of information

	**Mean (SD) score**		**Difference**		**Standardized**	Effect size**	**Paired t statistic**	p-value***
	**Pre**	**Post**	**Mean**	**SE**	response mean*			
Total score	40.2 (21.1)	31.7 (20.5)	-8.5	1.35	0.6	0.4	-6.3	<.0001
Uncertainty	45.0 (23.6)	37.2 (24.5)	-7.8	1.62	0.5	0.3	-4.8	<.0001
Informed	40.3 (23.8)	31.9 (24.0)	-8.4	1.74	0.5	0.4	-4.8	<.0001
Values clarity	44.2 (25.2)	32.1 (24.2)	-12.1	1.88	0.7	0.5	-6.4	<.0001
Support	37.2 (22.2)	29.4 (21.0)	-7.8	1.60	0.5	0.3	-4.9	<.0001
Effective decision	35.7 (22.1)	28.7 (20.8)	-7.0	1.46	0.5	0.3	-4.8	<.0001

*(pre-post)/SD (pre-post), **(pre-post)/SD (pre), ***Paired t-test.

SD; Standard deviation, SE; Standard error.

Pharmacists’ provision of information in this study, which is not a special intervention but a daily practice, had a much greater impact on the values clarity and informed subscales. On the other hand, the differences in the uncertainty, support, and effective decision subscales before and after pharmacists’ provision of information were not as large. A factor affecting the difference of improvement between these subscales is the content of the information provided by the pharmacists. Pharmacists generally provide treatment information with a focus on treatment efficacy and side effects [32]. However, patients would usually consider the influence of treatment on social aspects (e.g., family, partner, work, home duties, and social activity) as side effects as well [33]. Pharmacists may be better able to provide this information along with the medical aspects. The lack of a correlation between QOL and decisional conflict also indicated that patients recommended for a new chemotherapy regimen are facing problems that are not measured by QOL instruments.

Taken together, our results indicate that pharmacists’ provision of information can contribute to shared decision making. Although the pharmacist’s role and responsibility as part of the cancer care team is developing and differs slightly for each country, decisional conflict has the potential to become a new aspect of pharmacist intervention. Unfortunately, the concept of shared decision making is not widely recognized in Japan. In order to improve decisional conflict and facilitate shared decision making, further studies are needed to determine the information that patients require in order to improve the quality of the information provided by pharmacists.

### Limitations

We acknowledge several limitations in our study. First, we were unable to examine criterion-related validity because there is no alternative scale to measure decisional conflict in Japanese. Second, our study included patients in different chemotherapy settings as well as patients with various types of cancer. Different treatment regimens and cancer types may influence the extent or the change of decisional conflict. Third, we assessed decisional conflict immediately before and after the pharmacists’ provision of information to exclude other factors affecting patients’ decisional conflict without investigating the appropriateness of assessing the influence of pharmacists’ provision of information immediately following the pharmacist-patient interaction.

## Conclusions

The Japanese version of the DCS appears to be valid and reliable as a measure for Japanese patients with cancer. We found that the information provided by pharmacists’ may decrease decisional conflict among patients with cancer who were recommended a new chemotherapy regimen. Further parallel group comparison studies are needed to confirm the influence of the information provided by pharmacists.

## Competing interests

The authors declare no competing interests.

## Authors’ contributions

TK designed this study, assisted in analysis, and drafted the manuscript. KA was involved in the design of the study and assisted in drafting the manuscript. TY performed all analyses, was involved in the design of the study design, and assisted in editing the manuscript. HS, YS, and MK participated in the study design. HT, TA, and SU provided oversaw the drafting of the manuscript. All authors read and approved the final manuscript.

## Authors’ information

TK is a pharmacist and researcher at Tokyo University of Pharmacy and Life Sciences. KA is a Japanese Society of Pharmaceutical Health Care and Sciences (JSPHCS)-certified Oncology Pharmacist and JSPHCS-certified Senior Oncology Pharmacist. TY is a professor at Tohoku University Graduate School of Medicine, as well as a biostatistician and methodologist in clinical oncology research.

## Pre-publication history

The pre-publication history for this paper can be accessed here:

http://www.biomedcentral.com/1472-6947/13/50/prepub

## References

[B1] Thorne S Oliffe JL Stajduhar KI Communicating shared decision-making: Cancer patient perspectives Patient Educ Couns 2013 90 3 291 296 10.1016/j.pec.2012.02.018 22464665

[B2] Stacey D Samant R Bennett C Decision making in oncology: a review of patient decision aids to support patient participation CA Cancer J Clin 2008 58 5 293 304 10.3322/CA.2008.0006 18755939

[B3] Kassirer JP Incorporating patients' preferences into medical decisions N Engl J Med 1994 330 26 1895 1896 10.1056/NEJM199406303302611 8196734

[B4] Silvestri G Pritchard R Welch HG Preferences for chemotherapy in patients with advanced non-small cell lung cancer: descriptive study based on scripted interviews BMJ 1998 317 7161 771 775 10.1136/bmj.317.7161.771 9740561 PMC28665

[B5] Rothenbacher D Lutz MP Porzsolt F Treatment decisions in palliative cancer care: patients' preferences for involvement and doctors' knowledge about it Eur J Cancer 1997 33 8 1184 1189 10.1016/S0959-8049(97)00034-8 9301440

[B6] Wood ME Fama TA Ashikaga T Muss HB Discrepancy between preference and actual adjuvant therapy for breast cancer Clin Breast Cancer 2010 10 5 398 403 10.3816/CBC.2010.n.053 20920985

[B7] O'Connor AM Drake ER Wells GA Tugwell P Laupacis A Elmslie T A survey of the decision-making needs of Canadians faced with complex health decisions Health Expect 2003 6 2 97 109 10.1046/j.1369-6513.2003.00215.x 12752738 PMC5060179

[B8] Keeney RL Decision analysis: an overview Oper Res 1982 30 5 803 838 10.1287/opre.30.5.803 10298708

[B9] O'Connor AM Validation of a decisional conflict scale Med Decis Making 1995 15 1 25 30 10.1177/0272989X9501500105 7898294

[B10] User Manual - Decisional Conflict Scale http://decisionaid.ohri.ca/eval_dcs.html

[B11] Gaston CM Mitchell G Information giving and decision-making in patients with advanced cancer: a systematic review Soc Sci Med 2005 61 10 2252 2264 10.1016/j.socscimed.2005.04.015 15922501

[B12] Craft PS Burns CM Smith WT Broom DH Knowledge of treatment intent among patients with advanced cancer: a longitudinal study Eur J Cancer Care (Engl) 2005 14 5 417 425 10.1111/j.1365-2354.2005.00601.x 16274462

[B13] Jefford M Tattersall MH Informing and involving cancer patients in their own care Lancet Oncol 2002 3 10 629 637 10.1016/S1470-2045(02)00877-X 12372725

[B14] Mills ME Sullivan K The importance of information giving for patients newly diagnosed with cancer: a review of the literature J Clin Nurs 1999 8 6 631 642 10.1046/j.1365-2702.1999.00296.x 10827609

[B15] Vogel BA Bengel J Helmes AW Information and decision making: patients' needs and experiences in the course of breast cancer treatment Patient Educ Couns 2008 71 1 79 85 10.1016/j.pec.2007.11.023 18191933

[B16] Slingsby BT Decision-making models in Japanese psychiatry: transitions from passive to active patterns Soc Sci Med 2004 59 1 83 91 10.1016/j.socscimed.2003.10.006 15087145

[B17] Horikawa N Yamazaki T Sagawa M Nagata T Changes in disclosure of information to cancer patients in a general hospital in Japan Gen Hosp Psychiatry 2000 22 1 37 42 10.1016/S0163-8343(99)00042-0 10715502

[B18] Sekimoto M Asai A Ohnishi M Nishigaki E Fukui T Shimbo T Imanaka Y Patients' preferences for involvement in treatment decision making in Japan BMC Fam Pract 2004 5 1 10.1186/1471-2296-5-1 15053839 PMC375530

[B19] Watanabe Y Takahashi M Kai I Japanese cancer patient participation in and satisfaction with treatment-related decision-making: A qualitative study BMC Public Health 2008 8 77 10.1186/1471-2458-8-77 18302800 PMC2291463

[B20] Horne R Hankins M Jenkins R The Satisfaction with Information about Medicines Scale (SIMS): a new measurement tool for audit and research Qual Health Care 2001 10 3 135 140 10.1136/qhc.0100135 11533420 PMC1743429

[B21] Aikens JE Piette JD Diabetic patients' medication underuse, illness outcomes, and beliefs about antihyperglycemic and antihypertensive treatments Diabetes Care 2009 32 1 19 24 10.2337/dc08-1533 18852334 PMC2606823

[B22] Bowskill R Clatworthy J Parham R Rank T Horne R Patients' perceptions of information received about medication prescribed for bipolar disorder: implications for informed choice J Affect Disord 2007 100 1–3 253 257 17174406 10.1016/j.jad.2006.10.018

[B23] Dohler N Krolop L Ringsdorf S Meier K Ko YD Kuhn W Schwalbe O Jaehde U Task allocation in cancer medication management - integrating the pharmacist Patient Educ Couns 2011 83 3 367 374 10.1016/j.pec.2011.03.002 21482061

[B24] Beaton DE Bombardier C Guillemin F Ferraz MB Guidelines for the process of cross-cultural adaptation of self-report measures Spine (Phila Pa 1976) 2000 25 24 3186 3191 10.1097/00007632-200012150-00014 11124735

[B25] Barnett AG van der Pols JC Dobson AJ Regression to the mean: what it is and how to deal with it Int J Epidemiol 2005 34 1 215 220 15333621 10.1093/ije/dyh299

[B26] Quinten C Coens C Mauer M Comte S Sprangers MA Cleeland C Osoba D Bjordal K Bottomley A Baseline quality of life as a prognostic indicator of survival: a meta-analysis of individual patient data from EORTC clinical trials Lancet Oncol 2009 10 9 865 871 10.1016/S1470-2045(09)70200-1 19695956

[B27] Kobayashi K Takeda F Teramukai S Gotoh I Sakai H Yoneda S Noguchi Y Ogasawara H Yoshida K A cross-validation of the European Organization for Research and Treatment of Cancer QLQ-C30 (EORTC QLQ-C30) for Japanese with lung cancer Eur J Cancer 1998 34 6 810 815 10.1016/S0959-8049(97)00395-X 9797690

[B28] Koedoot CG De Haes JC Heisterkamp SH Bakker PJ De Graeff A De Haan RJ Palliative chemotherapy or watchful waiting? A vignettes study among oncologists J Clin Oncol 2002 20 17 3658 3664 10.1200/JCO.2002.12.012 12202667

[B29] Koedoot N Molenaar S Oosterveld P Bakker P de Graeff A Nooy M Varekamp I de Haes H The decisional conflict scale: further validation in two samples of Dutch oncology patients Patient Educ Couns 2001 45 3 187 193 10.1016/S0738-3991(01)00120-3 11722854

[B30] Keating NL Beth Landrum M Arora NK Malin JL Ganz PA van Ryn M Weeks JC Cancer patients' roles in treatment decisions: do characteristics of the decision influence roles? J Clin Oncol 2010 28 28 4364 4370 10.1200/JCO.2009.26.8870 20713872 PMC2954135

[B31] Degner LF Kristjanson LJ Bowman D Sloan JA Carriere KC O'Neil J Bilodeau B Watson P Mueller B Information needs and decisional preferences in women with breast cancer JAMA 1997 277 18 1485 1492 10.1001/jama.1997.03540420081039 9145723

[B32] Liekweg A Westfeld M Jaehde U From oncology pharmacy to pharmaceutical care: new contributions to multidisciplinary cancer care Support Care Cancer 2004 12 2 73 79 10.1007/s00520-003-0539-4 14530957

[B33] Carelle N Piotto E Bellanger A Germanaud J Thuillier A Khayat D Changing patient perceptions of the side effects of cancer chemotherapy Cancer 2002 95 1 155 163 10.1002/cncr.10630 12115329

